# Epigenetic Regulation of Oxidative Stress in Ischemic Stroke

**DOI:** 10.14336/AD.2015.1009

**Published:** 2016-05-27

**Authors:** Haiping Zhao, Ziping Han, Xunming Ji, Yumin Luo

**Affiliations:** ^1^Cerebrovascular Diseases Research Institute, Xuanwu Hospital of Capital Medical University, Beijing 100053, China; ^2^2Department of Neurosurgery, Xuanwu Hospital of Capital Medical University, Beijing 100053, China; ^3^Center of Stroke, Beijing Institute for Brain Disorders, Beijing 100053, China

**Keywords:** Stroke, brain, epigenetics, oxidative stress

## Abstract

The prevalence and incidence of stroke rises with life expectancy. However, except for the use of recombinant tissue-type plasminogen activator, the translation of new therapies for acute stroke from animal models into humans has been relatively unsuccessful. Oxidative DNA and protein damage following stroke is typically associated with cell death. Cause-effect relationships between reactive oxygen species and epigenetic modifications have been established in aging, cancer, acute pancreatitis, and fatty liver disease. In addition, epigenetic regulatory mechanisms during stroke recovery have been reviewed, with focuses mainly on neural apoptosis, necrosis, and neuroplasticity. However, oxidative stress-induced epigenetic regulation in vascular neural networks following stroke has not been sufficiently explored. Improved understanding of the epigenetic regulatory network upon oxidative stress may provide effective antioxidant approaches for treating stroke. In this review, we summarize the epigenetic events, including DNA methylation, histone modification, and microRNAs, that result from oxidative stress following experimental stroke in animal and cell models, and the ways in which epigenetic changes and their crosstalk influence the redox state in neurons, glia, and vascular endothelial cells, helping us to understand the foregone and vicious epigenetic regulation of oxidative stress in the vascular neural network following stroke.

Oxidative stress is crucial in the pathogenesis of neurodegenerative disorders [1], and the relevant mechanisms of oxidative neuronal death especially mitochondrial dysfunction [2] in ischemic stroke have been extensively studied. More importantly, a recent study showed that androgens were neuroprotective when oxidative stress levels were minimal, but exacerbated oxidative stress damage in immortalized rat dopaminergic neuronal cells when oxidative stress levels were elevated [3]. This indicated that oxidative stress was not only strongly implicated in the progression of cell death, but could also define the neuroprotective or neurotoxic properties of other drugs following stroke [3].

Oxidative stress represents an imbalance between the elevated production of reactive oxygen species (ROS)/reactive nitrogen species (RNS) and missing antioxidants, leading to oxidative modifications of proteins, lipids, and DNA [4-9]. Epigenetics have been reported to be profoundly involved in oxidative stress responses, and cause-effect relationships have been established between ROS and epigenetic modifications in aging, cancer, acute pancreatitis, and non-alcoholic fatty liver disease [10-15]. Moreover, epigenetic regulatory mechanisms, especially the role of histone deacetylase inhibition during stroke recovery, have been summarized in several reviews focusing mainly on apoptosis, necrosis, and neuroplasticity [16-26]. However, the role of oxidative stress-induced epigenetic regulation in vascular neural networks following stroke has not been sufficiently explored.

Epigenetic mechanisms, referring to heritable changes in gene expression without changing the DNA sequence, mainly include DNA methylation, histone modification, and microRNAs (miRNAs), which specifically modulate the expression levels of single genes and functional gene networks [27-28]. In this review, we emphasize the epigenetic events resulting from oxidative stress in experimental stroke models *in vivo* and *in vitro*, and how epigenetic changes influence the redox state in vascular neural networks, with implications for the discovery of more sensitive and specific therapeutic targets based on a combination of antioxidant and epigenetic regulatory strategies for stroke.

## Oxidative stress regulate DNA methylation in stroke

CpG island methylation is a well-characterized epigenetic change that generally regulates global and specific gene expression by transcription inhibition [29]. That is mediated by DNA methyltransferases (DNMTs), which are abundant in the brain, includes Dnmt 3a and Dnmt 3b (*de novo* methylation), as well as Dnmt 1 (maintains methylation) [30]. In addition, gene silencing mediated by DNA methylation also contains the interactions of protein-DNA and protein-protein, with the primary recruitment of methyl-CpG-binding-domain (MBD) family includes MBD1-4 and MeCP2 (methyl-CpG-binding protein 2) and subsequent combination of histone-modifying enzymes, that together raises chromatin condensation and deactivation [31]. In this section, we summarize the changes in global and gene-specific methylation following ischemic stroke *in vivo* and oxidative stress *in vitro*, and especially the related epigenetic mechanisms induced by ROS/RNS and hyperhomocysteinemia (HHcy).

### ROS/RNS induced DNA methylation following stroke

The generation of ROS/RNS (e.g. H_2_O_2_, nitric oxide) directly modify cytosine chemically, with oxidative conversion of 5-methylcytosine (5-mC) to 5-hydroxymethylcytosine (5-hmC), inhibiting the binding of Dnmt1 and MBP to DNA, therefore changes its methylation pattern [32-34]. 5hmC level is increased in blood cell DNA from patients with acute ischemic stroke [35]. Moreover, peroxides also cause nucleobases modification like 5-chlorocytosine, which mimics 5-mC and induce improper Dnmt1 methylation within CpG sequences, inducing gene silence [36, 37]. These evidences provide a mechanistic link between oxidative stress and epigenetic changes via chemical DNA modifications and altering DNA-protein interactions.

It’s noted that global DNA methylation in neural cells was changed by ischemia or oxidative stress, and DNMT inhibitors could alleviate ischemia or oxidative stress-induced neural injury. *In vivo*, the global DNA methylation was significantly increased in infarcted tissue in model of cerebral ischemia [38,39]. And DNMT inhibitors conducted neuroprotection, treatment with the broad-spectrum DNMT inhibitor 5-aza-2′-deoxycytidine (5-aza-dC) and zebularine [38], as well as reduced levels of Dnmt1 in postmitotic neurons in transgenic mice [39], could alleviate cerebral ischemic injury. Except for DNMTs, the expression of MBD-family proteins is altered orderly within the hippocampus: MBD3 expression was significantly reduced 3 h after ischemia, while MBD2 expression was increased by 6 h after ischemia, and MBD1 and MeCP2 levels were both elevated by 24 h after ischemia [40]. *In vitro*, treatment with H_2_O_2_ for 1 h increased the global DNA methylation level in SH-SY5Y human neuroblastoma cells, however, long-term treatment (72 h) had the opposite effect, along with decreased expressions of Dnmt 1, Dnmt 3a and Dnmt 3b [41]. Furthermore, H_2_O_2_ treatment in SH-SY5Y cells for 1 h increased the DNA-binding activities of nuclear factor (NF)-κB and SP1/3, while 5-aza-dC pretreatment resulted in increased NF-κB DNA-binding activity [41]. In addition, DNMT inhibitors 5-azacytidine and 5-aza-dC reduced photodynamic-treatment, a therapy based on photosensitizer-mediated oxidative cytotoxicity, induced necrosis of glial cells [42]. Conversely, knockdown of a critical enzyme for DNA demethylation, ten-eleven translocation methylcytosine dioxygenase (Tet1), notably increased the H_2_O_2_-induced apoptosis of cerebellar granule cells [43].

Gene-specific hypermethylation also plays a vital role in the vulnerability to ischemic stroke. For instance, oxygen glucose deprivation (OGD) induced raised methylation in thrombospondin 1 (THBS1) promoter and consequently decreased gene expression in murine cerebral endothelial cells, while reoxygenation led to the opposite effect [44]. Additionally, increased methylation at promoter of angiotensin II type 2 receptor (AT2R) and resultant gene depression in the developing brain augmented the vulnerability of brain hypoxic-ischemic injury in the neonate, which is reversed by 5-aza-dC [45]. Besides, gestational hypoxia causes epigenetic repression of glucocorticoid receptor (GR) gene expression in the developing brain, resulted from the increased DNA methylation, decreased binding of transcription factors early growth response protein (EGR1) and Sp1 to GR gene promoters, therefore, enhances brain vulnerability to hypoxic-ischemic injury in neonatal rats [46]. In addition, clinical study showed that obesity and ischemic stroke modulate the methylation levels of KCNQ1 (potassium channel, voltage gated KQT-like subfamily Q, member 1) and WT1 (Wilms tumor 1) in white blood cells [47], and weight loss intervention program changed the methylation patterns of two stroke-related genes KCNQ1 and WT1 (Wilms tumor 1) in obese stroke patients [48]. Moreover, DNMTs not only catalyze DNA methylation, but also is involved in the removal of amino groups [49]. Taken together, the global and gene-specific methylation following stroke is multifunctional, and influences the susceptibility to brain lesion. Although the protection against cerebral ischemia of DNMT inhibitors which is aiming at the global DNA hypermethylation is promising, and functional study of gene-specific methylation following stroke is increasing, the oxidative-stress-related epigenetic mechanisms remain understood.

**Figure 1.**
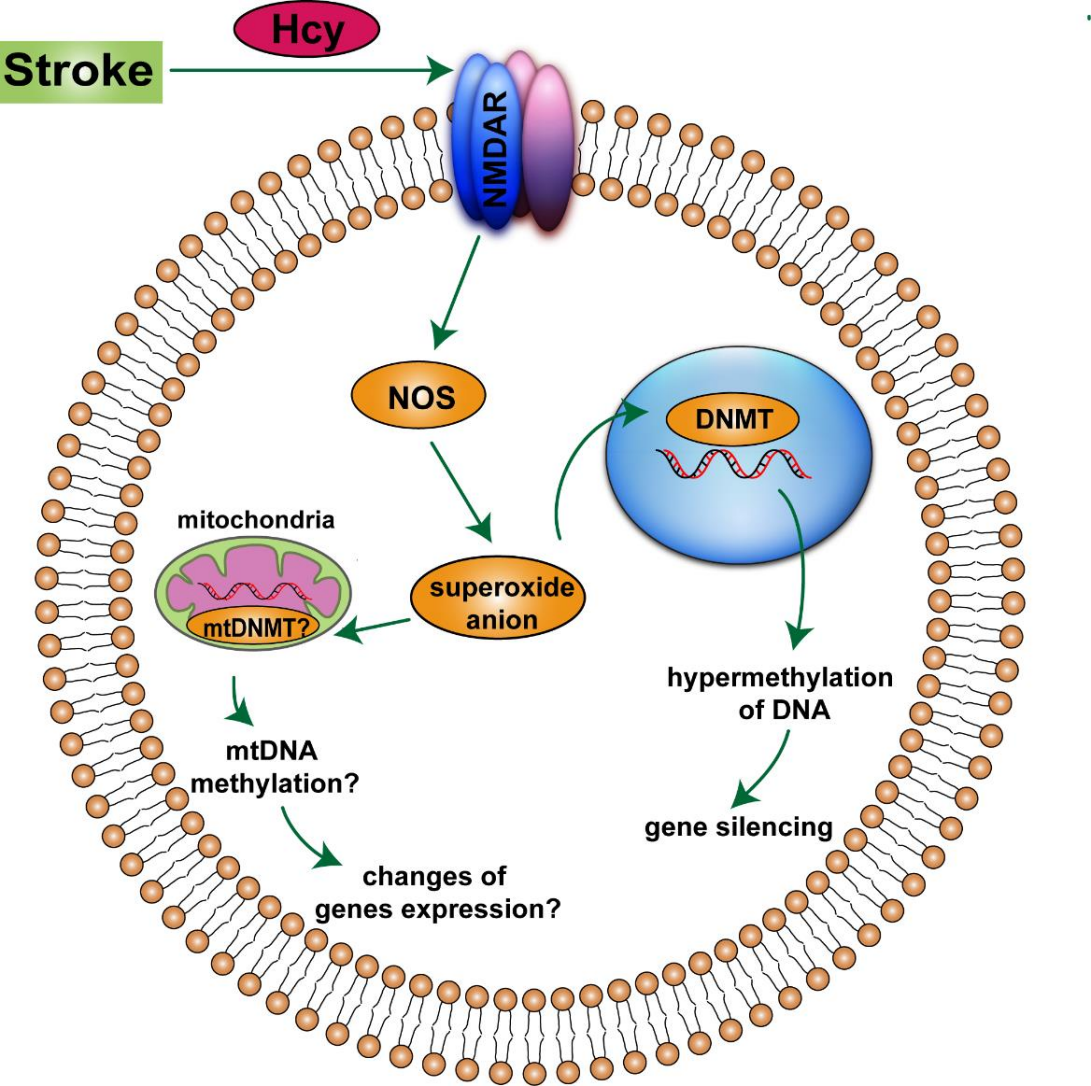
Schematic representation of the proposed mechanisms of hyperhomocysteinemia (HHcy) triggering epigenetic modifications following stroke. Hcy, homocysteine. NMDAR, N-methyl D-aspartate receptors. NOS, nitric oxide synthase. DNMT, DNA methyltransferase. mtDNMT, mitochondrial DNA methyltransferase.

### HHcy induced DNA methylation in stroke

Methylenetetrahydrofolate reductase (MTHFR) is important in DNA methylation, involving in the formation of methyl group donor S-adenosylmethionine. MTHFR deficiency-induced hyperhomocysteinemia (HHcy) could result in protein and lipoperoxidation, and further endothelial and neuronal degeneration and carotid artery plaques formation that may lead to an increased risk of ischemic stroke [50,51], and the genetic and epigenetic mechanisms are both involved [52-54]. Animals administered a folate/methyl-deficient diet showed global hypermethylation in the brain [55]. And clinical study showed that CpG A is a potential epigenetic marker in mediating serum folate and vitamin B12 to contribute to ischemic stroke [56]. Understanding HHcy-related epigenetics during brain ischemia may help in the discovery of sensitive biomarkers and in developing new therapeutic approaches for stroke.

N-methyl D-aspartate receptors (NMDAR)-mediated oxidative stress is the key pathway to the HHcy-related epigenetic regulation of vascular neural network following stroke. Endothelial cells and neurons contain NMDAR that have a high-affinity binding for Hcy, binding to the glutamate site of NMDAR1 initiates nitric oxide synthase (NOS) and generation of superoxide anions [57-60]. Hcy treatment was shown to increase the levels of DNMT1 and DNMT3a, but decrease DNMT3b levels in mouse brain endothelial cells, inducing mitochondrial toxicity and endothelial dysfunction [61]. In addition, HHcy inhibited the growth of arterial endothelial cells through altering promoter DNA methylation and inducing transcriptional repression of fibroblast growth factor-2 involving G-protein pathway [62]. Similar to endothelial cells, epigenetic alterations including increased levels of SAM and increased DNMT3a and DNMT3b activities were induced by HHcy in vascular smooth muscle cells, resulting in DNA hypermethylation and consequent gene silencing [63]. In contrast to vessel, primary cultures of astrocytes exposed to HHcy leaded to reduce total DNMT activity and a remarkable reduction in protein levels of DNMT 3b, accompanied by global DNA hypomethylation notably [64]. The discrepant pattern of global methylation and DNMTs activity surveyed in these investigations may suggest that the effects of HHcy on genomic DNA methylation are cell specific.

In addition to this nuclear mechanism, mitochondrial epigenetics (mito-epigenetics) are also involved in HHcy-related bone remodeling and skeletal muscle weakness [65, 66]. Mitochondrial DNA methylation has been demonstrated in humans and other mammalians and DNMT activity was present in mitochondria, and mitochondrial DNA was less methylated than nuclear DNA [67]. Recent studies of epigenetics along with the discovery of histone-like proteins in mitochondria indicate exciting new areas for mito-epigenetics. Because mitochondria is pivotal following ischemia-reperfusion injury through generating excessive ROS and hence damaging neural cells, Hcy-related mito-epigenetics during brain ischemia represents a promising area for stroke therapy (Fig. 1).

## Histone modification induced by oxidative stress following stroke

Histones wrap around DNA to form the nucleosome, and a variety of histone-modifying enzymes change the DNA conformation, leading to repositioning of nucleosomes, activating or preventing transcription [68]. The modifications are largely reversible and allow dynamic gene expression changes in response to the cellular environment. And modulation of histone-modifying enzymes has produced interesting results in stroke models, which has been reviewed recently [16-26], and we therefore focus on oxidative stress-induced histone modifications in stroke, and the influence of these modifications on gene transcription in the brain.

### Histone acetylation modulate oxidative stress following stroke

Histone acetylation/deacetylation is linked to transcriptional activation/depression, and is modified by histone acetyltransferases (HATs) and histone deacetylases (HDACs) respectively [69-72]. Given the clinic application of HDAC inhibitors (HDACi) in cancer treatment [73-80], and the promising result of HDACi in preclinical and clinical studies of stroke [81-86], the reuse of HDACi in stroke clinic should be expedited. Moreover, HDACi increase the acetylation of many non-histone proteins including hormone receptors, chaperone proteins, and transcription factors, thus modifying their activity or function [74]. In this part, we summarize the specific changes in HDAC proteins following stroke, and the function of pan-HDAC inhibition and distinctive HDAC isoforms involved in oxidative stress in cerebral ischemia.

Gene-expression mapping of the HDAC isoforms (classes I, II, and IV) under normal condition and following stroke has demonstrated their distinct regional, cellular and subcellular localizations and discrete substrates [87-89]. Under normal condition, HDACs are located mainly in neurons and mature oligodendrocytes; following stroke, the expression feature of the HDACs was changed [88, 89]. *In vivo*, HDAC1-2 was decreased in the ischemic core area, but increased in neurons of the penumbra in the subventricular zone and cortex, and in glial cells in the subcortical white matter following 45 min MCAO. HDAC1 was bright and encircled in the capillaries of contralateral tissue. HDAC2 is strongly expressed in the astrocyte end-feet in the hippocampus and cortex [88]. *In vitro*, HDAC1-3 expression levels were upregulated after 60 min of OGD in all glial cell nuclei and astrocyte processes, with HDAC3 being the most strongly upregulated [88]. Another study identified HDAC3 and HDAC6 as probable regulators of neurotoxicity in ischemic stroke, implying that therapeutic approaches aiming at specific HDAC subtype may be considered [89]. Given the toxicity of pan-HDACi towards a host of CNS cell types and the opposing effects of HDACi on unique cell types [90], identification of HDAC isoforms involved in stroke and of those responsible for specifically mediating the beneficial function of pan-HDAC inhibition is needed to conquer this barrier.

HDACs participate in the progress of oxidative stress following stroke by altering the functions of histone or non-histone proteins through posttranslational deacetylation. Pre-treatment with the HDACi trichostatin A (TSA) was demonstrated to protect against OGD in primary cortical neurons by enhancing histone acetylation in the promoter region of gelsolin, a pivotal mediator of actin-filament assembly-disassembly, in dose- and time-dependent manners [91]. The transcription factors Sp1 and Nrf-2 participate in the antioxidant-responsive function of HDACi. TSA augmented transcription factor Sp1 acetylation, and associated loss of DNA binding and its downstream gene expression of Catalase, MnSOD and p21 waf1/cip1, mitigating the glutamate analog homocysteate-induced oxidative neuronal death *in vitro* and 3-nitroproprionic acid-caused oxidative neuronal death *in vivo* [92]. Nrf2 is a critical mediator of antioxidant-responsive genes in stroke, and pharmacologic inhibition of HDAC could not protect Nrf2-deficient mice against cerebral ischemia. Moreover, HDACi reduced expression of the Keap1, induced Keap1/Nrf2 dissociation and Nrf2 nuclear translocation, upregulating proteins downstream of Nrf2, including heme oxygenase 1 (HO-1), glutamate-cysteine ligase catalytic subunit (GCLC), and NAD(P)H:quinone oxidoreductase 1 (NQO1) in neuronal cultures and brain tissue [93]. Resveratrol leads to Nrf-2 protein acetylation thereby providing cell protection against cerebral ischemia through modulation of sirtuin activity, a nicotinamide adenosine dinucleotide-dependent histone deacetylase [94]. These results suggested that Nrf2 activation might be a vital mechanism by which HDACi provides neuroprotection. In addition, the HDACi valproic acid and TSA can inhibit photodynamic-therapy-induced necrosis and apoptosis of satellite glial cells [42]. HDACi can protect against oxidative neuronal death induced by peroxide addition or glutathione depletion [95]. Several hydroxamate-based HDACi can protect neurons from oxidative stress via an HDAC-independent mechanism, involving the *in situ* formation of hydroxamate-iron complexes that catalyze the decomposition of H_2_O_2_ in a manner reminiscent of catalase [96].

Importantly, HDAC subtypes play different roles in oxidative stress following stroke. Ischemia/reperfusion (I/R) reduced phosphorylation at Ser 394 of HDAC2, and weakened the HDAC2-FOXO3a reciprocity in mouse brain tissue. Moreover, H_2_O_2_ also reduced the HDAC2-FOXO3a interaction in cerebellar granule neurons, resulting in elevated histone H4K16 acetylation in the promoter region of p21 and upregulated its expression. This study revealed novel epigenetic regulation of FOXO3a-mediated gene expression during oxidative stress-induced neuronal cell death, which could be developed therapeutically [97]. In addition, H_2_O_2_ treatment induced translocation of HDAC4 from the cytoplasm into the nucleus in cultured cortical neurons, where it interacted physically with peroxisome proliferator-activated receptor-γ and repressed its transcriptional activity and inhibited its pro-survival activity, thus regulating neuronal death [98]. HDAC5 and HDAC4 were markedly reduced in both cerebral ischemia/reperfusion injury and OGD model, and NADPH oxidase-reduced HDAC4 and HDAC5 accelerates cerebral ischemia injury via increasing the expression and release of high mobility group box-1 protein (HMGB1) [99]. A selective and robust increase of HDAC6 expression associated with homocysteic acid-induced oxidative neuronal injury was demonstrated by real-time polymerase chain reaction, and inhibition of HDAC6 can promote the neuronal survival [100]. In accordance with its cytoplasmic localization, the function of HDAC6 inhibition appears to be transcription-independent. Particularly, the selective inhibition of HDAC6 avoids cell death associated with pan-HDAC inhibition, defining HDAC6 as a latent non-toxic therapeutic target for alleviating CNS injury against oxidative stress-induced neurodegeneration.

Despite numerous studies, the mechanisms responsible for the protection of HDACi remain to be adequately illuminated, while the specific subtypes of HDACs associated with ischemic stroke remain unclear. Investigating the effects of these types of modulation on oxidative stress-induced inhibition of synaptic plasticity in relation to stroke recovery will provide important mechanistic insights. Given that some small-molecule HDACi are currently in use in patients or clinical trials, HDACi represent promising treatment approach for stroke patients.

### Histone methylation and demethylation under oxidative stress in stroke

Histone methylation, modified by histone methyltransferases (HMTs), was always considered to be a permanent epigenetic modification [101-103], but the discovery of histone demethylases (HDMs) has changed this perception [104-106]. Although increasing numbers of HMTs and HDMs have been identified, their functions in the experimental stroke remain inadequately understood. In contrast to the neuroprotective effects of DNA methylation and histone acetylation inhibition, the role of histone methylation in transcriptional response following stroke remains intricate. In this section, we summarize the changes and functions of HMTs such as SUV39H1 and G9a, and HDMs such as JmjC-domain-containing histone demethylases (JHDMs) and lysine-specific demethylase 1 (LSD1) following stroke respectively, and the mechanism involved in relation to oxidative stress.

H3K4 HMT activity was recently shown to be decreased in astrocytes from middle-aged female rats compared to adult females of stroke [85]. And enhanced cell survival following ischemia in adult females was correlated with enriched H3K4me3 at the miR-17-20 cluster and VEGFa and subsequent greater VEGF protein expression and miR-20 mRNA expression [85]. However, no methylation difference was detected at the H3K36 position in neonatal hypoxic-ischemic brain injury [107]. A new study showed that inhibition of the repressive H3K9 HMTs SUV39H1 and G9a using either RNA interference or the specific blocker chaetocin improved neuronal survival in an OGD model, partly mediated by the increased H3K9ac in promoter regions of brain-derived neurotrophic factor and its active transcription [108].

The JHDMs and LSD1 are needed to demethylate histone H3 at Lys4 or Lys 9, two specific tags for epigenetic transcriptional activation. It was proved that JmjC domain uses Fe(II) and α-ketoglutarate as cofactors in an oxidative demethylation reaction via hydro-xymethyl lysine, indicated by that excessive Fe(II) or ascorbate can rescue H_2_O_2_-mediated impairment of histone demethylase activity [109]. The members of this family functions in brain development, however, their effect in stroke remains to be illuminated. In addition, the expression of LSD1 changes temporally and spatially following brain ischemia and reperfusion injury. The numbers of LSD1-positive cells in the DG and CA1 regions significantly increased as soon as 1 h post-ischemia, peaked at 6 h and day 3 respectively, suggesting that LSD1 may be involved in neural regeneration following stroke [110]. And it was proved that LSD1 plays an important role in silencing neuronal-specific genes in non-neuronal cells [111], also promoted long-term memory [112]. Importantly, LSD1 is a flavin-dependent amine oxidase, which could stimulate androgen-receptor-dependent transcription coverting oxygen to H_2_O_2_ [113, 114]. Given the involvement of JHDMs and LSD1 in oxidative response, future studies are needed to explore their significance in regulation of oxidative stress following stroke.

Interestingly, LSD1 acts as a component of various transcriptional co-repressor complexes rather than a free-functioning enzyme *in vivo* [115-119]. LSD1 participates in HDAC1/2-mediated deacetylation of H3K9Ac which is thought to precede the binding of CoREST, followed by LSD1-mediated H3K4me1/2 demethylation [120]. In addition, HDAC4 plays a central role in the rapid modification of histone methylation in response to variations in cardiac load in patients with heart failure [121]. The existence of crosstalk between histone modifications suggest that further investigations is required to clarify the function of LSD1 inhibition on histone methylation and the link between methylation changes and acetylation. However, the role of LSD1 remains largely unexplored. In addition, the potentially reversible modes of LSD1 inhibition that may alter LSD1 through mechanisms other than competitive inhibition of substrates are required to be explored, and specific molecular targets of LSD1 upon oxidative stress and how they are linked to the regulation of transcription are also needed to be identified.

### Histone phosphorylation-mediated neural necrosis upon oxidative stress in stroke

In addition to the methylation and acetylation of histone lysine residues, serine and threonine phosphorylation also occur during cerebral ischemia [122-124]. Phosphorylated Histone 2AX (γ-H2AX) occurs under oxidative stress and accumulates with progressive injury following stroke. The overactive glutamate receptor (GluR) after ischemia increases oxidative stress and evoked γ-H2AX in neurons, which was alleviated by pretreatment with the antioxidant. The generation of γ-H2AX following GluR activation corresponded to the increases observed following exposure to H_2_O_2_. These data suggest that insults not necessarily resulting in neuronal death may induce the DNA-damage-evoked chromatin modification, γ-H2AX, implicating histone alterations in determining neuronal vulnerability following neurological insults [122]. Glutamate-induced calcium influx in neurons activates the ERK1/2 and its downstream of MSK1/2/JIL-1, which increases phosphorylation of histone H3 at serine 28 (pH3S28), displace polycomb repressive complex 1 from chromatin, then activates Trithorax, leading to increased H3K4me3, resulting in cell necrosis by an unknown mechanism [123]. Oxidative stress induced enhanced pH3 at serine 10 (pH3S10) in mouse brain endothelial cells after OGD, resulting in cell death and pH3 interacted with IKKa in the nucleus. And IKKa siRNA treatment significantly reduced cell death and pH3 level after OGD, suggesting the crucial function of pIKKa and subsequent phosphorylation of histone H3 in response to oxidative stress in cell death after cerebral ischemia [124]. Together, these results indicated that phosphorylation of histone-mediated chromatin-modifying cascade was involved in neuronal necrosis following cerebral ischemia and oxidative stress. However, the mechanism of phosphorylation histone related with oxidative stress following stroke is rarely studied and need further elucidation.

### MiRNA-mediated regulation of oxidative stress following stroke

MiRNAs are approximately 22 nucleotides small RNA molecules, which negatively regulate the expression of the target genes post-transcriptionally. MiRNAs are involved in stroke risk factors including atherosclerosis, hypertension, atrial fibrillation, diabetes, and dyslipidemia. The function of miRNAs in the pathophysiology of stroke become one subject of recent researches [125], including miRNAs regulating apoptosis and autophagy of neurons, astrocytes, and cerebral vascular endothelial cells after stroke [126-130], though studies of their mechanisms in relation to oxidative stress are limited. In this section, we examine the relationships between miRNAs, oxidative stress, and epigenetic machinery.

The effect of oxidative stress on miRNA expression profile in mouse primary hippocampal neurons was studied. MiR-708 and miR-135b were significantly increased upon H_2_O_2_ stimulation, and their targets were related with DNA recombination, protein autophosphorylation, protein ubiquitination, and neurons development [131]. Cell death was reduced when miR-181a levels were reduced, and increased when miR-181a levels increased in N2a cells upon serum deprivation (SD) and oxidative stress; protection was associated with increased Bcl-2 protein [132]. Our group showed that miR-424 reduced oxidative stress in the cortex and protected against transient cerebral ischemia-reperfusion injury. MiR-424 treatment abrogated H_2_O_2_-induced lactate dehydrogenase leakage and increased manganese superoxide dismutase activity in neuronal cultures, and its protective effects against oxidative stress were reversed by Nrf-2 knockdown and superoxide dismutase (SOD) inhibition [133]. We also demonstrated that miR-23a-3p dose-dependently reduced H_2_O_2_-induced generation of nitric oxide and 3-nitrotyrosine, thus reversing the decreased activities of total SOD and manganese SOD in N2a neuroblastoma cells. Furthermore, miR-23a-3p suppressed oxidative stress and relieved cerebral ischemia-reperfusion injury [134]. In addition, vagus nerve stimulation was neuroprotective against cerebral ischemia-reperfusion injury and modulates redox status through activating neuronal and astrocyte α7n acetylcholine receptor and possibly associated with increased miR-210 expression [135].

Oxidative stress-induced endothelial dysfunction plays a pivotal role in ischemia-reperfusion injury. Recent evidence indicates that endothelial progenitor-cell-derived microvesicles can promote angiogenesis of endothelial cells. A hypoxia/reoxygenation model of human brain microvascular endothelial cells was produced by 6 h hypoxia and 24 h reoxygenation. Functionally, serum deprivation had beneficial effects on hypoxia/reoxygenation-exposed endothelial cells, whereas serum-deprived medium containing tumor necrosis factor-α (apoptotic stress) had detrimental effects. These results suggest that the serum-deprived and apoptotic-stress endothelial progenitor-cell-derived microvesicles were functionally different in terms of apoptosis and dysfunction via their RNAs, such as miR126 associated with ROS production and the phosphoinositide 3-kinase/endothelial NOS/nitric oxide pathway [136]. MiR-204 augmenting the susceptibility of RSC96 Schwann cells to H_2_O_2_-induced apoptosis through down-regulating the expression of neuritin, which act as a neurotrophin and play crucial role in plasticity and repair following nervous system injury. Therefore, low-level expression of miR-204 may create a suitable microenvironment for the nerves repair by lightening the transcriptional inhibition of neuritin transcription [137]. MiRNAs are currently undergoing evaluation for feasible clinical use as biomarkers for neurological diseases [138].

In another study, exosomes were used to deliver therapeutic mRNA/protein to treat cancer [139]. However, the delivery of therapeutic miRNAs for stroke treatment remains largely unexplored.

### Conclusions

Clinical and preclinical investigations suggest a critical relationship between oxidative stress and epigenetic mechanisms following stroke. Peroxide could induce global and gene-specific DNA methylation, and influences the susceptibility to brain ischemia. HDAC subtypes play different roles in oxidative stress following stroke by altering the functions of histone or non-histone proteins. In contrast, the role of histone methylation in transcriptional response following stroke remains intricate, but JHDMs and LSD1 are important in oxidative response, future studies are needed to explore their significance in stroke. Histone phosphorylation mainly mediated neural necrosis upon oxidative stress in stroke. MiRNAs are changed upon oxidative stress in the vascular neural networks and may connect the ischemic brain with other organs. However, although compelling, these findings raise new questions. The issues of whether epigenetic remodeling promotes susceptibility to oxidative stress insults following ischemia-reperfusion, and if epigenetic modifications occur in response to oxidative stress insults following ischemia-reperfusion remain to be answered. Given the redox state following stroke could influence the effect of neuroprotective drugs, the investigations of the function of oxidative stress on epigenetic modifications are critical for formulating effective individualized therapeutic approaches for stroke patients.
